# Using Machine Learning Techniques to Aid Empirical Antibiotic Therapy Decisions in the Intensive Care Unit of a General Hospital in Greece

**DOI:** 10.3390/antibiotics9020050

**Published:** 2020-01-31

**Authors:** Georgios Feretzakis, Evangelos Loupelis, Aikaterini Sakagianni, Dimitris Kalles, Maria Martsoukou, Malvina Lada, Nikoletta Skarmoutsou, Constantinos Christopoulos, Konstantinos Valakis, Aikaterini Velentza, Stavroula Petropoulou, Sophia Michelidou, Konstantinos Alexiou

**Affiliations:** 1 School of Science and Technology, Hellenic Open University, 26335 Patras, Greece; kalles@eap.gr; 2 IT Department, Sismanogleio General Hospital, 15126 Marousi, Greece; v_loupelis@sismanoglio.gr (E.L.); spetrop@sismanoglio.gr (S.P.); 3 Department of Quality Control, Research and Continuing Education, Sismanogleio General Hospital, 15126 Marousi, Greece; 4 Intensive Care Unit, Sismanogleio General Hospital, 15126 Marousi, Greece; sakagianni@sismanoglio.gr (A.S.); valakis@sismanoglio.gr (K.V.); sofimicheli@yahoo.gr (S.M.); 5 Microbiology Laboratory, Sismanogleio General Hospital, 15126 Marousi, Greece; mikroviologiko@sismanoglio.gr (M.M.); lettaskarm@yahoo.gr (N.S.); micr.fym@sismanoglio.gr (A.V.); 6 2nd Internal Medicine Department, Sismanogleio General Hospital, 15126 Marousi, Greece; malvinalada@gmail.com; 7 1st Internal Medicine Department, Sismanogleio General Hospital, 15126 Marousi, Greece; cgchrist@otenet.gr; 8 1st Surgery Department, Sismanogleio General Hospital, 15126 Marousi, Greece; knegro@otenet.gr

**Keywords:** antibiotic resistance, antimicrobial resistance, intensive care unit, ICU, machine learning, prediction, artificial intelligence, ML techniques

## Abstract

Hospital-acquired infections, particularly in the critical care setting, have become increasingly common during the last decade, with Gram-negative bacterial infections presenting the highest incidence among them. Multi-drug-resistant (MDR) Gram-negative infections are associated with high morbidity and mortality with significant direct and indirect costs resulting from long hospitalization due to antibiotic failure. Time is critical to identifying bacteria and their resistance to antibiotics due to the critical health status of patients in the intensive care unit (ICU). As common antibiotic resistance tests require more than 24 h after the sample is collected to determine sensitivity in specific antibiotics, we suggest applying machine learning (ML) techniques to assist the clinician in determining whether bacteria are resistant to individual antimicrobials by knowing only a sample’s Gram stain, site of infection, and patient demographics. In our single center study, we compared the performance of eight machine learning algorithms to assess antibiotic susceptibility predictions. The demographic characteristics of the patients are considered for this study, as well as data from cultures and susceptibility testing. Applying machine learning algorithms to patient antimicrobial susceptibility data, readily available, solely from the Microbiology Laboratory without any of the patient’s clinical data, even in resource-limited hospital settings, can provide informative antibiotic susceptibility predictions to aid clinicians in selecting appropriate empirical antibiotic therapy. These strategies, when used as a decision support tool, have the potential to improve empiric therapy selection and reduce the antimicrobial resistance burden.

## 1. Introduction

The rapid emergence of antibiotic-resistant infections during the last decade constitutes a worldwide problem with increasing health and economic costs [1]. As stated in a recently published European Centre for Disease Prevention and Control (ECDC) study, about 33,000 people die each year as a direct consequence of an infection due to bacteria resistant to antibiotics [2]. Healthcare associated infections (HAIs) account for the major burden of these multidrug-resistant infections, while last-line treatments, such as carbapenems and colistin, become less effective, eliminating the available therapeutic options [3].

Data from the European Antimicrobial Resistance Surveillance Network (EARS-Net) suggest that in 2015, Greece was among the countries with the greatest burden of infections due to antibiotic-resistant bacteria in the EU and European Economic Area (EEA) [4], with carbapenem- and colistin-resistant infections presenting the major problem [2,4,5]. The Hellenic Center for Disease Control and Prevention (HCDCP) in 2014 reported a mean incidence of 0.48 per 1000 patient-days, and a crude 28-day mortality rate of 34.4%, caused by carbapenem-resistant Gram-negative pathogens in acute care hospitals in Greece [6].

In our recent study [7], we compared the resistance levels of *Pseudomonas aeruginosa*, *Acinetobacter baumannii*, and *Klebsiella pneumoniae* isolates between the intensive care unit (ICU) and other facilities in two consecutive years (2017 and 2018), in one of the largest public tertiary hospitals in Greece, to implement more effective strategies for the reduction of multidrug resistance. By using the same antimicrobial susceptibility dataset from the Microbiology Laboratory, we proposed a methodology [8] that enables clinicians to select the most appropriate antibiotic based on statistically significant sensitivity results, which are specific for their own department. 

Many hospitals focus on early detection of serious infections, especially in ICUs. It has been shown that the earlier the proper antibiotic treatment starts, the lower the mortality rate [9,10]. From a clinical point of view, the detection of antimicrobial resistance before culture and sensitivity results are available will reduce the time required to take important actions, such as isolating the patient or initiating appropriate empirical therapy. 

Advances in artificial intelligence (AI) have transformed the healthcare innovation environment, contributing to improved health outcomes while reducing healthcare costs. AI is now calling to explore new possibilities in healthcare that were previously regarded as not feasible. For example, due to the digitization of health records, mining of unstructured medical data is now possible and, using this, clinicians can readily make various evidence-based decisions.

Machine learning (ML) techniques could be used to establish a clinical decision support system to aid clinicians to make effective choices. The scientific literature review shows promising results in the use of ML techniques in healthcare, particularly in antimicrobial resistance research [11,12,13,14]. In this article, we propose using ML techniques to predict antimicrobial resistance based only on data available in the hospital information system of the Microbiology Laboratory, such as the type of sample, Gram stain, and previous antibiotic susceptibility testing together with patient demographics (age/gender).

## 2. Methodology and Results

We analyzed, in a 2-year period (2017 and 2018), the data of the Microbiology Laboratory from ICU patients in a public tertiary hospital in Greece. The dataset of 23,067 instances contain the attributes of gender (binary), age (numerical), type of sample (categorical), Gram stain (binary), antibiotics (categorical), and finally the class attribute, which in our case is the antimicrobial susceptibility (binary). The samples examined were blood, tracheobronchial aspirates/ bronchoalveolar lavage fluid, urine, skin/wounds/soft tissue specimens, intravascular catheters, and pleural and peritoneal fluid. In the present study, clinical data of the patients, such as the source of infection acquisition (e.g., community or hospital acquired), and the presence of active infection or colonization, have not been included. The following table (Table 1) includes simple summary statistics of our dataset.

Among the many existing machine learning systems, we have chosen to use (in this study) the WEKA—Data Mining Software in Java Workbench [15]. It is one of the most popular open-source machine learning toolkits and contains a wide range of learning algorithms.

To assess the performance of the final model [16], some data must be set aside and not used during training so that we may compare what is known about these data to what our algorithms will predict. This is the test set. If we use all of our data to train a model, and then use the same data for testing, we run the risk of learning tiny details, which will be of little use with new data.

A good way to make the most of our data is to use all of our data for training as well as for testing, but not at the same time. To do this, we divide our data into a number of equal-sized subsets, called folds. For each fold, we remove it from the training set, build a model on the other folds, and then test on the withheld portion. If we have *k* folds, then this is called *k*-fold cross-validation. Cross-validation is widely regarded as a reliable way to assess the quality of results from machine learning techniques when data are all in one set. In our analysis, we have used 10-fold cross-validation.

To find the best classifier, we consider the following quantities, as reported by WEKA [17,18]:

(a) TP Rate: rate of true positives (instances correctly classified as a given class);
(b) FP Rate: rate of false positives (instances falsely classified as a given class);
(c) Precision: the proportion of instances that are truly of a class divided by the total instances classified as that class;
(d) Recall: the proportion of instances classified as a given class divided by the actual total in that class (equivalent to TP rate);
(e) F-Measure: a general indicator of the quality of the model;
(f) MMC: a correlation coefficient calculated from all four values of the confusion matrix.
(g) Area under the Receiver Operating Characteristics (ROC) curve (AUC): a graphical plot that illustrates the performance of a binary classifier system as its discrimination threshold is varied. The accuracy of the test depends on how well the test separates the group being tested into those with and without the disease in question. Accuracy is measured by the area under the ROC curve;
(h) The Precision-Recall Plot (PRC) plot shows the relationship between precision and sensitivity.

### 2.1. LIBLINEAR-L2-Regularized L1- and L2-loss Support Vector Classification (SVC)

LIBLINEAR is an open-source library for large-scale linear classification [19]. It supports logistic regression and linear support vector machines. 

Given training vectors $x_{i}\in R^{n},\;i=1,\ldots l$ in two classes, and a vector $y\in R^{l}$ such that $y_{i}=\left\{1,-1\right\}$, a linear classifier generates a weight vector w as the model. The decision function is

$$sgn\left(w^{T}x\right)$$

L2-regularized L2-loss SVC solves the following primal problem:

$$\underset{w}{\min}\frac{1}{2}w^{T}w+C\overset{l}{\underset{i=1}{\sum }}{\left(\max\left(0,\;1-y_{i}w^{T}x_{i}\right)\right)}^{2}$$

and its dual form is:

$$\underset{a}{\min}\;\frac{1}{2}a^{T}\bar{Q}a-e^{T}a$$

$$\mathrm{subject}\;\mathrm{to}\;0\leq a_{i}\leq U,\;i=1,\ldots l$$

where e is the vector of all ones, $\bar{Q}=Q+D,\;D$ is a diagonal matrix, and $Q_{ij}=y_{i}y_{j}x_{i}^{T}x_{j}$

$$\mathrm{For}\;L2\text{-}\mathrm{loss}\;\mathrm{SVC},\;U=\infty \;\mathrm{and}\;D_{ii}=\frac{1}{2C},\;\forall i$$

The results of applying this technique are shown in the following table (Table 2). 

### 2.2. LIBSVM C-Support Vector Classification

Support Vector Machines (SVMs) are a set of related supervised learning methods, which are popular for performing classification, regression, and other learning tasks. LIBSVM [20] is an integrated software for SVMs classification. One of the SVM formulations of LIBSVM is the C-Support Vector Classification. Given training vectors $x_{i}\in R^{n},\;i=1,\ldots l$, in two classes, and a vector $y\in R^{l}$ such that $y_{i}=\left\{1,-1\right\}$, C-SVC [21,22] solves the following primal optimization problem.

$$\underset{w,b,\xi }{\min}\;\frac{1}{2}w^{T}w+C\overset{l}{\underset{i=1}{\sum }}\xi_{i}$$

$$\mathrm{subject}\;\mathrm{to}\;y_{i}\left(w^{T}\phi \left(x_{i}\right)+b\right)\geq 1-\xi_{i},\;\xi_{i}\geq 0,\;i=1,\ldots l$$

where $\phi \left(x_{i}\right)$ maps $x_{i}$ into a higher-dimensional space, and C>0 is the regularization parameter. The corresponding dual form is:

$$\underset{a}{\min}\;\frac{1}{2}a^{T}\bar{Q}a-e^{T}a$$

$$\mathrm{subject}\;\mathrm{to}\;y^{T}a=0,\;0\leq a_{i}\leq C,\;i=1,\ldots l$$

where e is the vector of all ones, Q is an l by l positive semidefinite matrix,

$$Q_{ij}=y_{i}y_{j}\left(\phi {\left(x_{i}\right)}^{T}\phi \left(x_{j}\right)\right)$$

by using the primal-dual relationship, the optimal w satisfies

$$w=\overset{l}{\underset{i=1}{\sum }}y_{i}a_{i}\;\phi \left(x_{i}\right)$$

and the decision function is

$$sgn\;\left(w^{T}\phi \left(x_{i}\right)+b\right)=sgn\;\left(\overset{l}{\underset{i=1}{\sum }}y_{i}a_{i}\;\left(\phi {\left(x_{i}\right)}^{T}\phi \left(x\right)\right)+b\right)$$

The results of applying this technique are shown in the following table (Table 3).

### 2.3. Sequential Minimal Optimization (SMO)

Sequential Minimal Optimization (SMO) [23] is a simple algorithm that quickly solves the SVM quadratic programming (QP) optimization problem without extra matrix storage and without invoking an iterative numerical routine for each sub-problem. SMO chooses to solve, at every step, the smallest possible problem of optimization. The smallest possible problem of optimization involves two Lagrange multipliers for the standard SVM QP problem because the Lagrange multipliers must obey a linear constraint of equality. SMO selects two Lagrange multipliers to optimize together at each step, finds the optimal values for these multipliers, and updates the SVM to reflect the new optimal values [24].

The results of applying this technique are shown in the following table (Table 4).

### 2.4. Instance-Based Learning (k-Nearest Neighbors)

Instance-based learning approaches [25], such as the k-nearest neighbors (kNN) algorithm, adopt a straightforward approach to estimate real or discrete-valued target functions [26,27]. Predicting the output of a new input vector involves collecting and aggregating outputs from similar instances from the saved training data. Unlike many other techniques that create only one local approximation to the target function, an important advantage of instance-based algorithms is that the model can build a new approximation to the target function for each new query instance. This gives instance-based algorithms the ability to capture very complicated relationships between attributes and outputs. If the target variable depends only on a few of the attributes, this can cause very similar instances to be predicted at a large distance [28,29].

The results of applying this technique are shown in the following tables (Table 5 and Table 6).

### 2.5. J48

The classification algorithm J48 is the implementation of the Quinlan C4.5 algorithm [30]. C4.5 uses the gain ratio for feature selection and to construct the decision tree. The C4.5 algorithm for building decision trees is implemented in WEKA as a classifier called J48. C4.5 can be referred to as the statistic classifier. It handles both continuous and discrete features. The C4.5 algorithm is widely used because of its quick classification and high precision. 

The results of applying this technique are shown in the following table (Table 7).

### 2.6. Random Forest

The random forest machine learner is a meta-learner, meaning, consisting of many individual learners (trees). The random forest uses multiple random tree classifications to vote on an overall classification for the given set of inputs. In general, each individual machine learner vote is given equal weight. In Breiman’s later work [31], this algorithm was modified to perform both unweighted and weighted voting. The forest chooses the individual classification that contains the most votes. 

A random forest is a classifier consisting of a collection of tree-structured classifiers $\left\{h\left(x,\Theta_{\kappa }\right),\;\kappa =1,\ldots \right\}$ where the $\left\{\Theta_{\kappa }\right\}$ are independent, identically distributed random vectors, and each tree casts a unit vote for the most popular class at input x.

The results of applying this technique are shown in the following table (Table 8).

### 2.7. RIPPER 

RIPPER [32] is an acronym for repeated incremental pruning to produce error reduction. Classes are analyzed in increasing size and use incremental reduced-error pruning to produce an initial set of rules for the class. This adds an extra stop condition that depends on the description length (DL) of the examples and the set of rules [33]. The formula of description length (DL) takes into account the number of bits required to send a set of examples with respect to a set of rules, the number of bits required to send a rule with k conditions, and the number of bits needed to send the integer *k*—times an arbitrary factor of 50 percent, to compensate for potential inconsistency in the attributes. 

The results of applying this technique are shown in the following table (Table 9).

### 2.8. Multilayer Perceptron (MLP)

A classifier that uses backpropagation to learn a multi-layer perceptron to classify instances. MLP is an artificial neural network model that maps input data to a set of suitable outputs [15,16]. This type of neural network is known as a supervised network because, in order to learn, it needs a desired output. The goal of this type of network is to create a model that correctly maps the input to the output using historical data, so that when the desired output is unknown, the model can be used to generate the output. This consists of multiple layers of nodes in a directed graph, as its name suggests, with each layer fully connected to the next. The network can be built by hand or set up using a simple heuristic. The nodes in this network are all sigmoid.

The results of applying this technique are shown in the following table (Table 10).

According to Table 11, considering the weighted average values, it can be seen that Multilayer perceptron and J48 (C4.5) algorithms outperform other models, with respect to the ROC area, with values of 0.726 and 0.724, respectively. RIPPER is the best at F-measure value with a value of 0.678.

## 3. Discussion

It is well known that the ICU environment presents the greatest burden of multidrug-resistant infections among hospital wards. As time is critical, rapid confirmation of the pathogen and its susceptibility profile warrants tailored and effective therapy and increases the chance of a favorable outcome [9,10]. 

Recently, machine learning (ML) algorithms have been proposed to predict antibiotic resistance phenotypes based on genomic features analysis with promising results [34,35]. The implementation of these techniques is nevertheless more expensive and complicated compared to standard antibiotic susceptibility testing. 

The aim of the present study was to investigate whether readily available susceptibility data from the Microbiology Department, together with simple demographic data, could be used in an algorithm to predict antibiotic resistance and guide antibiotic empirical prescription in critically ill patients in a timely and cost-effective manner.

The methods proposed in this paper will allow us to anticipate culture and sensitivity results from the Microbiology Laboratory. The early detection of patients at high risk for resistance to one or more families of antibiotics may lead to useful knowledge of the patient and hospital ecosystem, and subsequent better management of the healthcare resources. Firstly, it could support the physician in selecting the appropriate empiric therapy as an immediate benefit. On the other hand, targeted empirical therapy may limit antibiotic misuse and, over time, reduce the prevalence of antibiotic-resistant bacteria. In addition, patients with multidrug-resistant infections could be isolated to prevent potential outbreaks of resistant bacteria, and thus, avoid inadvertent spread to other ICU patients. Such intervention will result in lower mortality, lower workload, lower hospital costs, and a decrease in infections during ICU stays.

Our methodology is based solely on data of the Microbiology Laboratory that already exists in the hospital’s Laboratory Information System. Similar studies [11,12,13,14] use ML techniques to predict antimicrobial susceptibility with many more attributes, including clinical data of the patients, and other useful information related to the domain examined. The purpose of our study is to present a low-cost approach that may be used in any ICU, requiring only the existence of an elementary information system of the Microbiology Laboratory (sometimes that could be a simple database). Among the various ML models examined, the best performance achieved was 0.726, which means that we can predict susceptibility to a specific antibiotic with an accuracy of 72.6%, based solely on the source of the specimen and the presumed site of infection, the Gram stain of the pathogen, and previous susceptibility data. Of course, the performance of the techniques that we present in this study will be substantially improved if the antimicrobial susceptibility datasets include the patient’s clinical information as well. Additionally, we also note that, had this research been conducted with the view of actually providing information that would be integrated into the clinician’s everyday practice, a more professional data processing package would have been required and, substantially, more studies would have to be conducted to boost the statistical confidence of our results. For example, a more thorough line of investigation could have aimed to assess (and, subsequently, control) the degree of bias possibly introduced due to the existence of multiple samples from a given patient, since this raises the possibility that patients with one resistant organism (or with an organism with resistance to a specific antibiotic) will have other resistant organisms (or the same resistance mechanisms in multiple species of bacteria) due to shared (unmeasured) risk factors, and/or horizontal gene transfer. While there do exist techniques, like boosting, which can reduce bias, we expect to examine them in future work and, at this stage, as it stands, we consider our results promising from the point of view of demonstrating the apparent feasibility and relative ease with which readily available data can be utilized to provide rule-of-thumb actionable information to time-pressed clinicians. Thus, the key message from our investigation is that, even with the most elementary data, one can take several steps towards improving the ICU performance.

## 4. Materials and Methods

This study examines the performance of eight machine learning models based on data of the Microbiology Laboratory from ICU patients in a public tertiary hospital in Greece. It is a general 12-bed ICU with mixed medical and surgical cases.

### 4.1. Samples-Source of Isolates

During the two years (January 2017–December 2018), a total of 888 clinical samples from 345 ICU patients were included in this study and processed by the Microbiology Laboratory according to established protocols [36,37,38]. The types of samples examined and their percentages are presented in Section 2 (Table 1). Blood cultures were incubated in the BacT/Alert system (bioMerieux). Isolation and identification of pathogens were carried out according to classical microbiological procedures [39]. 

### 4.2. Antimicrobial Susceptibility Data

Antimicrobial susceptibility testing was performed by the MicroScan system (Siemens), according to Clinical and Laboratory Standards Institute (CLSI) guidelines [40,41] and the results were confirmed, when necessary, using a gradient minimum inhibitory concentration (MIC) determining method following the manufacturer’s guidelines (e.g., the E-test bioMerieux, Sweden). MICs of colistin retested via microtiter plates (SensiTestColistin, Liofilchem). Sensitivity and resistance breakpoints for the antibiotics were determined according to CLSI interpretive criteria [40,41] and for tigecycline and fusidic acid, according to Eucast ones [42]. *Escherichia coli* ATCC 25922 strain, *Pseudomonas aeruginosa* ATCC 27853, and *Staphylococcus aureus* ATCC 29213 and ATCC 25923 were used as quality control strains for susceptibility testing. 

The phenotypic detection of the production of extended-spectrum beta-lactamases (ESBL) was performed by the double-disk synergy test (DDST), according to CLSI guidelines [40]. Metallo-beta-lactamases (MBL) and carbapenemases (KPC) were detected phenotypically by (a) the modified odge test [40], (b) the combined disk test, with a meropenem (MER) disk alone, a MER disk plus phenyl boronic acid (PBA), a MER disk plus EDTA, and a MER disk plus PBA and EDTA, as described by Tsakris et al. [43], and c) the NG CARBA 5 immunochromatographic assay, targeting KPC-, NDM-, VIM-, and IMP-type and OXA-48-like carbapenemases, following the manufacturer’s guidelines (data presented at 29th European Congress of Clinical Microbiology & Infectious Diseases (ECCMID) 2019 [44]. *P. aeruginosa* strains were tested phenotypically for MBL, either by a combined disk test using the imipenem (IPM) disk, and IPM plus EDTA, as described by Yong et al. [45], or by an IPM-EDTA double-disk synergy test (DDST), as described by Lee et al. [46]. All strains that phenotypically produced more than one or no carbapenemases, the oxa producers, and all those tested with NG CARBA 5, were subject to PCR for bla_NDM_, bla_VIM_, bla_KPC_, and bla_OXA-48_ genes. They were also examined for the presence of the plasmid-mediated *mcr*-1 gene for colistin-resistance (data presented at 28th and 29th ECCMID 2019 [44,47]).

### 4.3. Bacterial Pathogens and Antibiotics

The resistance for *P. aeruginosa* was measured based on the following antibiotics: amikacin, aztreonam, cefepime, ceftazidime, ciprofloxacin, colistin, gentamicin, imipenem, meropenem, doripenem, piperacillin/tazobactam, tobramycin, and levofloxacin. *P. aeruginosa* strains presented the highest resistance rates to gentamycin (57.97%) and cefepime (56.67%), followed by fluoroquinolones (55.11%) and carbapenems (55.02%) [7]. 

The resistance for *A. baumannii* was measured based on the following antibiotics: amikacin, ampicillin/sulbactam, cefepime, cefotaxime, ceftazidime, ciprofloxacin, colistin, gentamicin, imipenem, levofloxacin, meropenem, minocycline, tobramycin, trimethoprim/sulfamethoxazole, tetracycline, and tigecycline. A high resistance rate of over 80% of *A. baumannii* isolates to most classes of antibiotics was identified, with the lowest resistance rates reported to colistin (53.37%) [7].

The resistance for *K. pneumoniae* was measured based on the following antibiotics: amikacin, amoxicillin/clavulanic acid, ampicillin/sulbactam, cefepime, cefotaxime, cefoxitin, ceftazidime, cefuroxime, ciprofloxacin, colistin, ertapenem, gentamicin, imipenem, meropenem, piperacillin/tazobactam, tetracycline, tobramycin, trimethoprim/sulfamethoxazole, levofloxacin, moxifloxacin, and tigecycline. The highest resistance rate was reported to older beta lactams/beta lactamase inhibitors (amp/sulb 85.98%), fluoroquinolones (up to 83.04%), carbapenems (up to 81.44%) and third generation cephalosporins (up to 81.61%) [7]. 

The resistance for *Achromobacter xylosoxidans* was measured based on the following antibiotics: amikacin, aztreonam, ceftazidime, gentamicin, imipenem, meropenem, minocycline, piperacillin/tazobactam, tobramycin, levofloxacin, and trimethoprim/sulfamethoxazole. 

The resistance for *Enterobacter aerogenes* was measured based on the following antibiotics: amikacin, amoxicillin/ clavulanic acid, ampicillin/sulbactam, cefepime, cefotaxime, cefoxitin, ceftazidime, cefuroxime, ciprofloxacin, colistin, ertapenem, gentamicin, imipenem, levofloxacin, meropenem, moxifloxacin, piperacillin/tazobactam, tetracycline, tobramycin, and trimethoprim/sulfamethoxazole. 

The resistance for *Enterobacter cloacae* was measured based on the following antibiotics: amikacin, amoxicillin/ clavulanic acid, ampicillin/sulbactam, aztreonam, cefepime, cefotaxime, cefoxitin, ceftazidime, ceftriaxone, cefuroxime, ciprofloxacin, colistin, ertapenem, gentamicin, imipenem, levofloxacin, meropenem, moxifloxacin, piperacillin/tazobactam, tetracycline, tobramycin, and trimethoprim/sulfamethoxazole.

The resistance for *E. coli* was measured based on the following antibiotics: amikacin, amoxicillin/clavulanic acid, ampicillin, ampicillin/sulbactam, aztreonam, cefepime, cefotaxime, cefoxitin, ceftazidime, ceftriaxone, cefuroxime, ciprofloxacin, colistin, ertapenem, gentamicin, imipenem, levofloxacin, meropenem, moxifloxacin, piperacillin/tazobactam, tetracycline, tobramycin, and trimethoprim/sulfamethoxazole. 

The resistance for *Proteus mirabilis* was measured based on the following antibiotics: amikacin, amoxicillin/clavulanic acid, ampicillin, ampicillin/sulbactam, aztreonam, cefepime, cefotaxime, cefoxitin, ceftazidime, ceftriaxone, cefuroxime, ciprofloxacin, ertapenem, gentamicin, imipenem, levofloxacin, meropenem, moxifloxacin, piperacillin/tazobactam, tobramycin, and trimethoprim/sulfamethoxazole. 

The resistance for *Stenotrophomonas maltophilia* was measured based on the following antibiotics: ceftazidime, levofloxacin, minocycline, and trimethoprim/sulfamethoxazole.

The resistance for *Enterococcus faecalis* was measured based on the following antibiotics: amoxicillin/clavulanic acid, ampicillin, ciprofloxacin, daptomycin, gentamicin500, levofloxacin, linezolid, pristinamycin, quinupristin/dalfopristin, rifampin, streptomycin 1000, teicoplanin, tetracycline, and vancomycin. 

The resistance for *Enterococcus faecium* was measured based on the following antibiotics: amoxicillin/clavulanic acid, ampicillin, ciprofloxacin, daptomycin, gentamicin 500, levofloxacin, linezolid, pristinamycin, quinupristin/dalfopristin, rifampin, streptomycin 1000, teicoplanin, tetracycline, and vancomycin. 

The resistance for *S. aureus* was measured based on the following antibiotics: ceftaroline, ciprofloxacin, clindamycin, daptomycin, erythromycin, fusidic acid, gentamicin, levofloxacin, linezolid, oxacillin, penicillin, pristinamycin, quinupristin/dalfopristin, rifampin, teicoplanin, tetracycline, tobramycin, trimethoprim/sulfamethoxazole, and vancomycin. Methicillin-resistance (MRSA) was found in 21.12% of the total *S. aureus* isolates.

Our research focuses only on the antibiotics mentioned above since there is an adequate number of samples for these for deducing reliable conclusions for the models that were examined. The incidence of multidrug (MDR) or extensively drug resistant (XDR) bacteria was not examined in the present study. In the present study, bacteria were assigned as sensitive or resistant against each antibiotic tested. As mentioned in Section 4.2, phenotypical detection of ESBL and KPC production was performed, but these results were not included in the dataset used in ML models.

## 5. Conclusions

In this paper, we evaluated a collection of very popular learning classifiers on an ICU antimicrobial susceptibility dataset. The best results achieve an F-measure of 0.678 with the RIPPER algorithm and an ROC area of 0.726, with the Multilayer perceptron classifier. The experimental results demonstrate that, especially, the Multilayer perceptron and J48 (C4.5) algorithms are suitable models for ICU antimicrobial susceptibility data sets with the evaluation of ROC Area results. The decision to use one of these techniques as an assistant depends mainly on whether the ICU places a premium on the accuracy or explainability, though there do exist approaches that attempt to bridge these preferences. Given the fact that the algorithms presented contain only a few variables, retrieved solely from the Microbiology Department without adjuvant clinical data, the best performances achieved were not high enough to characterize our techniques widely applicable. Despite limitations of the study, our primary goal was to take advantage of these data using ML techniques and possibly create an inexpensive ancillary tool to aid the clinician in identifying patients carrying antibiotic-resistant bacteria and guide proper therapy with greater confidence in situations where there is significant uncertainty and a crucial decision needs to be taken.

In future work, we will focus on enriching our datasets with clinical attributes as well as investigating the configurations, which would improve the algorithms’ performances.

## Figures and Tables

**Table 1:** Simple summary statistics of the dataset.

Age (Years)		Gender	Gram Stain	Class
Mean	61.68	Male (44%)	Positive (16.20%)	Resistant (51.20%)
St.Dev.	19.66	Female (56%)	Negative (83.80%)	Sensitive (48.80%)
Range	80			
Type of Samples				
Blood (7.38%)		Tracheobronchial (60.90%)	Urine (20.40%)	Peritoneal (2.01%)
Tissue (5.29%)		Catheters (3.76%)	Pleural (0.26%)	

**Table 2:** Detailed accuracy by class of LIBLINEAR (10-fold cross-validation).

Measure	*TP* Rate	FP Rate	Precision	Recall	F-Measure	MCC	ROC Area	PRC Area	Class
	0.593	0.456	0.577	0.593	0.585	0.137	0.568	0.550	R
	0.544	0.407	0.560	0.544	0.552	0.137	0.568	0.527	S
Weighted Avg.	0.569	0.432	0.569	0.569	0.569	0.137	0.568	0.539	

**Table 3:** Detailed accuracy by class of LIBSVM (10-fold cross-validation).

Measure	*TP* Rate	FP Rate	Precision	Recall	F-Measure	MCC	ROC Area	PRC Area	Class
	0.752	0.433	0.646	0.752	0.695	0.325	0.660	0.613	R
	0.567	0.248	0.686	0.567	0.621	0.325	0.660	0.600	S
Weighted Avg.	0.662	0.343	0.665	0.662	0.659	0.325	0.660	0.607	

**Table 4:** Detailed accuracy by class of SMO (10-fold cross-validation).

Measure	*TP* Rate	FP Rate	Precision	Recall	F-Measure	MCC	ROC Area	PRC Area	Class
	0.787	0.470	0.638	0.787	0.705	0.329	0.659	0.611	R
	0.530	0.213	0.704	0.530	0.605	0.329	0.659	0.602	S
Weighted Avg.	0.662	0.344	0.670	0.662	0.656	0.329	0.659	0.607	

**Table 5:** Detailed accuracy by class of lB1 (1 nearest neighbor) (10-fold cross-validation).

Measure	*TP* Rate	FP Rate	Precision	Recall	F-Measure	MCC	ROC Area	PRC Area	Class
	0.727	0.448	0.630	0.727	0.675	0.283	0.682	0.656	R
	0.552	0.273	0.658	0.552	0.600	0.283	0.682	0.666	S
Weighted Avg.	0.641	0.363	0.644	0.641	0.639	0.283	0.682	0.661	

**Table 6:** Detailed accuracy by class of lB5 (5 nearest neighbor) (10-fold cross-validation).

Measure	*TP* Rate	FP Rate	Precision	Recall	F-Measure	MCC	ROC Area	PRC Area	Class
	0.726	0.432	0.638	0.726	0.679	0.298	0.711	0.687	R
	0.568	0.274	0.664	0.568	0.612	0.298	0.711	0.717	S
Weighted Avg.	0.649	0.355	0.651	0.649	0.647	0.298	0.711	0.702	

**Table 7:** Detailed accuracy by class of J48 (10-fold cross-validation).

Measure	*TP* Rate	FP Rate	Precision	Recall	F-Measure	MCC	ROC Area	PRC Area	Class
	0.765	0.427	0.653	0.765	0.705	0.345	0.724	0.696	R
	0.573	0.235	0.699	0.573	0.630	0.345	0.724	0.733	S
Weighted Avg.	0.671	0.333	0.676	0.671	0.668	0.345	0.724	0.714	

**Table 8:** Detailed accuracy by class of random forest (10-fold cross-validation).

Measure	*TP* Rate	FP Rate	Precision	Recall	F-Measure	MCC	ROC Area	PRC Area	Class
	0.674	0.396	0.641	0.674	0.657	0.279	0.703	0.681	R
	0.604	0.326	0.638	0.604	0.621	0.279	0.703	0.717	S
Weighted Avg.	0.640	0.362	0.640	0.640	0.639	0.279	0.703	0.698	

**Table 9:** Detailed accuracy by class of RIPPER (10-fold cross-validation).

Measure	*TP* Rate	FP Rate	Precision	Recall	F-Measure	MCC	ROC Area	PRC Area	Class
	0.735	0.380	0.670	0.735	0.701	0.358	0.699	0.653	R
	0.620	0.265	0.691	0.620	0.653	0.358	0.699	0.694	S
Weighted Avg.	0.679	0.324	0.680	0.679	0.678	0.358	0.699	0.673	

**Table 10:** Detailed accuracy by class of Multilayer perceptron (10-fold cross-validation).

Measure	*TP* Rate	FP Rate	Precision	Recall	F-Measure	MCC	ROC Area	PRC Area	Class
	0.706	0.380	0.661	0.706	0.683	0.327	0.726	0.706	R
	0.620	0.294	0.668	0.620	0.643	0.327	0.726	0.743	S
Weighted Avg.	0.664	0.338	0.664	0.664	0.663	0.327	0.726	0.724	

**Table 11:** Weighted average values of F-Measure and Receiver Operating Characteristics (ROC) area for all methods (10-fold cross-validation).

Technique	F-Measure	ROC Area
LIBLINEAR	0.569	0.568
LIBSVM	0.659	0.660
SMO	0.656	0.660
kNN-5	0.647	0.711
J48	0.639	0.724
Random Forest	0.639	0.703
RIPPER	0.678	0.699
Multilayer perceptron	0.663	0.726
